# Low-Thujone *A. absinthium* L. (Wormwood) Essential Oils and Extracts with Potential Antioxidative/Prooxidant Activity

**DOI:** 10.3390/molecules31101551

**Published:** 2026-05-07

**Authors:** Asta Judžentienė, Jurga Būdienė

**Affiliations:** Department of Organic Chemistry, Center for Physical Sciences and Technology, Saulėtekio Avenue 3, LT-10257 Vilnius, Lithuania; jurga.budiene@ftmc.lt

**Keywords:** *Artemisia absinthium*, essential oils, aqueous/methanolic extracts, GC/MS, HPLC/DAD/TOF, TPC (total phenolic content), amperometry, H_2_O_2_ scavenging potential, prooxidant ability

## Abstract

Nowadays, the global demand for medicinal plants, including *A. absinthium* L. (wormwood), has increased considerably, leading to significant pressure on their wild populations and the biodiversity of ecosystems. Consequently, the rates of exploitation may exceed those of natural regeneration. This destructive process can be reduced by cultivating plants with the desired secondary metabolites by transferring them from their natural habitats. The present study investigates phytochemistry and the potential antioxidative/prooxidant activity of low-thujone *A. absinthium* plants. The chemical composition of wormwood extracts and essential oils (EOs) was determined by HPLC/DAD/TOF and GC/MS techniques, respectively. *Trans*-Sabinyl acetate (59.6 ± 10.1%) predominated in the wormwood EOs, while the content of toxic *trans*-thujone was negligible (1.2 ± 0.5%). Eighteen acids, such as fumaric, ascorbic, succinic, quinic, malic, gallic, benzoic, (neo/iso)chlorogenic, (di)ferulic, caffeic, etc., were found in 50% methanolic wormwood extracts. Additionally, (epi)catechin, astragalin, diosmetin, piceatannol-3’-*O*-glucoside, quercetin-3-*O*-glucoside, quercetin-3-*O*-rhamnoside-7-*O*-glucoside, hesperidin, apigenin-7-*O*-glucoside, baicalin, 5,7,3′-trihydroxy-3,6,4′,5′-tetramethoxyflavone and rutin were tentatively identified in the extracts. Total phenolic content was found 412.82 ± 11.10 mg/L (of gallic acid equivalent) in *A. absinthium* methanolic extracts. Using conventional spectroscopic methods, the antioxidant activity (DPPH radicals scavenging) was determined to be 0.83 ± 0.06 mmol/L (TROLOX equivalent) in the wormwood essential oil. ABTS^●+^ and DPPH^●^ scavenging activity means, 3.485 ± 0.07 (TROLOX, mmol/L) and 6.48 ± 0.25 (TROLOX, mmol/L) were revealed for *A. absinthium* methanolic extracts. Less commonly used methods, electrochemical tests showed the presence of oxidizable compounds with characteristic E_pa_ values of 0.38 and 0.61 V. Moreover, hydrogen peroxide scavenging tests were performed. The largest quantity of peroxide (31.86 ± 0.1 μmol/L) was formed in the wormwood boiling infusions (at pH = 7.2). As the presence of toxic and neurotoxic thujone isomers is undesirable, therefore, the search for low- or thujone-free plants from natural populations that exhibit biological activity (i.e., antioxidant/prooxidant) is of great importance.

## 1. Introduction

*Artemisia* (including about 500 taxa/species) is one of the largest genera in the tribe *Anthemideae* Cass. (f. *Asteraceae*), growing in both hemispheres, but distributed mainly in the northern temperate regions [1,2,3,4,5]. Asia has the greatest concentration of species, with 150 accessions for China, 174 in the former Soviet Union, about 50 reported for Japan, 35 species of the genus found in Iran, and about 30 in Italy [4,5].

**Figure 1:**
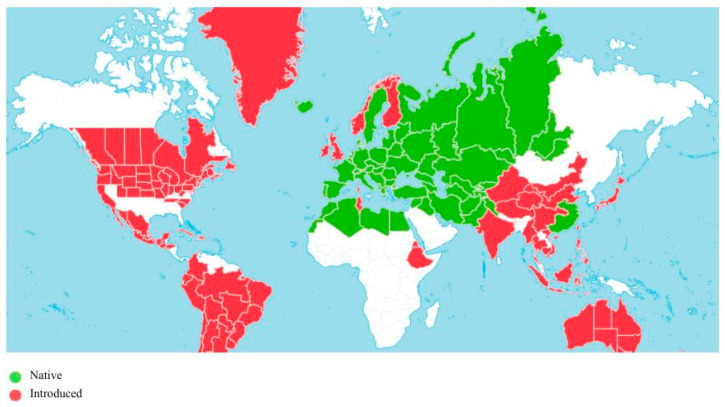
A visual representation of the global distribution of *A. absinthium* L. plants. The map was created using data from Plants of the World Online (POWO) [1] by Canva (https://www.canva.com).

*A. absinthium* L. is a native perennial shrub in Europe from Iceland and Scotland to the Mediterranean regions, found in northern India, northern Africa (Egypt, Morocco, Lebanon–Syria, Libya, Western Sahara), and introduced to both American continents, Great Britain, Ethiopia, India, Vietnam, N. Zealand and Australia [1]. The global distribution of *A. absinthium* L. plants as a native or alien species is presented in Figure 1.

The common name of *A. absinthium* L. is “wormwood”, reflecting its traditional use as an anthelmintic for expelling parasitic worms. A literature survey on *A. absinthium* phytochemistry [2,3,4,5,6,7,8,9,10,11,12,13,14,15,16,17,18,19,20,21,22,23,24,25,26,27,28,29,30,31,32,33,34,35,36,37,38,39,40,41,42] revealed that the plant extracts contain various compounds, such as terpenoids [2,3,4,5,6,7,8,9,11,14,17,20,21,22,23,24,25,26,27,28,29,30,31,32,33,35,36,39,40,42], organic acids [3,5,6,7,8,9,10,12,13,15,16,17,24,34,37], lactones [2,3,5,6,7,13,18,19,24,38], flavonoids [2,3,5,6,7,8,9,10,13,15,16,17,24,37,38,41], coumarins [6], sterols [6,12,13], resins [5,6], carotenoids [6], saponins [14] and tannins [2,5,6,14,17,24]. Due to the numerous different bioactive constituents, wormwood has been used as a remedy for the treatment of helminthiasis [2,6,13,14,16,19], fever [2,6,8], anemia [6,7], insomnia [6], bladder diseases [6], wounds [6,7,15], gastrointestinal ailments [2,4,5,6,7,8,10,19], to reduce pain during childbirth and menstrual problems or to cause abortion in the ethno- and traditional Asian and European medicine [2,5]. Dried herbs are used as a pest repellent to keep insects and mice away.

Currently, numerous other biological activities have been evaluated and confirmed by scientific research [42,43,44,45,46,47,48,49,50,51,52,53,54,55,56,57,58,59,60]. Wormwood extracts exhibit analgesic [6,7,15], antibacterial [2,4,6,7,8,10,11,12,15,16,20,40,42], cardiac stimulant [2,7,17], antidiabetic [3,7,15,54], antihypertensive [15,19], antipyretic [3,6,7,15,19,43], antifungal [4,6,7,8,12,16,20,43], hepatoprotective [2,3,6,7,17,44,45,56], neuroprotective [2,3,6,7,8,12,17], antidepressant [6,7], cytotoxic [3,6,7,9,22,41,44,46,48,49], phytotoxic [4,8,11,13,14,15,18,20,25,26,38], immuno-stimulatory [6,7], antispasmodic [2], anthelmintic [2,5,6,7,13,14,16,17,20,43,59,60], anti-inflammatory [2,3,6,7,9,48,54,56], antioxidant [2,3,6,7,8,9,10,12,16,17,20,22,31,35,43,47,48,51,52,53] and memory-improving properties [2,6,15,17]. Additionally, wormwood allelopathic effects on weeds [38,57] and against gastrointestinal parasites in domestic animals were reported [14,59,60]. In the food industry, wormwood is used as a spice herb for flavoring in some alcoholic beverages and vermouth-type wines; it is an important ingredient of the liquor absinthe. *A. absinthium* is successfully used in the cosmetic industry [6,24]. The species is an object of biotechnological research [45,48]. *A. absinthium* is cultivated in many countries, such as Colombia, Brazil, USA, Kashmir Valley (India), Britain and Southern Europe [1,6,24,38,61,62]. Wormwood breeding is easy due to its good adaptability, high drought tolerance, resistance to weeds and pests, and tolerance to a wide range of temperatures, including cold winters. 

The main reasons for the industrial production of wormwood are its medicinal properties and its use as a flavoring ingredient in alcoholic beverages. Due to hazardous thujone, preparation of *A. absinthium* is not recommended without professional medical guidance. The use of thujone in food and alcoholic beverages is restricted in many regions, particularly in the European Union and the United States. According to European Union regulations (Annex II of Directive 88/388/EEC (EEC, 1988)) on flavorings, the following maximum levels for thujone (*α*- and *β*-) in foodstuffs, beverages or other food ingredients are 0.5 μg/kg (with some exceptions). Thujone may not be added as such to food. Moreover, thujone is not authorized for use as a flavoring substance in the USA. In general, in many cases, the presence of toxic and neurotoxic thujone isomers (*cis*- and *trans*-) is undesirable; therefore, the search for low- or thujone-free plants with biological activity is very important.

The aim of the present study was to determine the chemical composition and total phenolic content (TPC) of the EOs and extracts (aqueous and alcoholic (methanol:water, 50:50, *v*/*v*)) of *A. absinthium* L. (wormwood), in addition to evaluating their antioxidant activity (AA) and prooxidant properties. A wide range of compounds exhibit AA; therefore, a single method for determining AA is not sufficient. For this reason, two different techniques, such as conventional spectrophotometric radical (ABTS^●+^ and DPPH^●^ assays) and less commonly used electrochemical tests (differential pulse voltammetry (DPV)) will be applied in this research. Moreover, it is known that many polyphenolic compounds present in the medical herbs could exhibit dual AA and prooxidant (hydrogen peroxide formation) activities [63]. For this reason, the prooxidant activity of *A. absinthium* various water extracts will be evaluated.

## 2. Results

### 2.1. Chemical Ccomposition of A. absinthium L. (Wormwood) EOs, Using GC/FID and GC/MS

**Figure 2:**
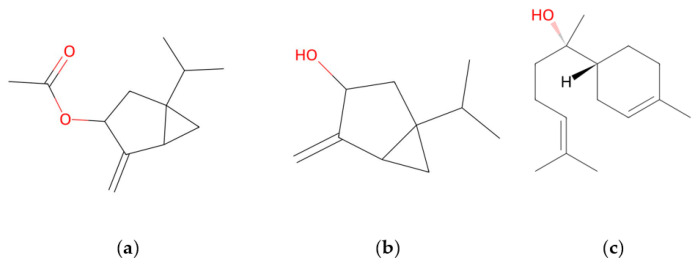
Chemical structures of *trans*-sabinyl acetate (**a**), *trans*-sabinol (**b**) and *epi*-*α*-bisabolol (**c**).

The yield of wormwood EOs (*v*/*w*, on a dry weight basis) was found to be in average 2.6%. The oil has a pungent specific odor and is dark orange-brown in color. Full compositional data of wormwood EOs are presented in Table 1. Totally, 40 compounds were identified in the oils, comprising averagely 95.5%. *Trans*-Sabinyl acetate (59.6 ± 10.1%), *trans*-sabinol (5.9 ± 2.1%) and *epi-α*-bisabolol (4.1 ± 1.1%) were found to be the main constituents in the EO*s*, while content of toxic *trans*-thujone was insignificant (1.2 ± 0.5%). Chemical structures of main compounds are presented in Figure 2.

### 2.2. Chemical Composition of A. absinthium L. Extracts

A minimum of 30 compounds were identified tentatively in the wormwood leaf and inflorescence aqueous and alcoholic (methanol:water, 1:1, *v*/*v*) extracts. Identification of compounds was performed by DAD and TOF (in positive and/or negative ionization mode) (Table 2). Four phenolic acids (chlorogenic, neochlorogenic, isochlorogenic A and B) were detected under both ionization conditions. In addition, the identification of certain constituents was achieved by their standards.

### 2.3. Total Phenolic Content (TPC) and Antioxidant Activity (AA) of A. absinthium EOs and Extracts

**Figure 3:**
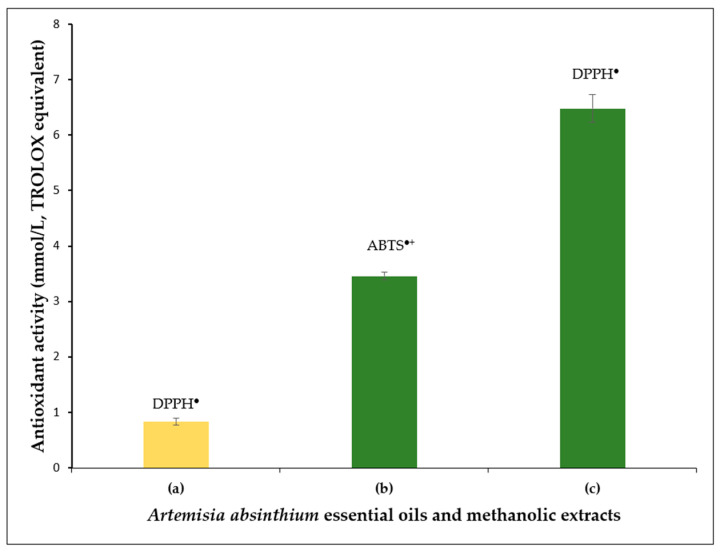
Scavenging activity (mmol/L, TROLOX) of *A. absinthium* EO (**a**) and methanolic extracts (**b**,**c**).

TPC was determined to be 412.82 ± 11.10 (mg/L, GAE, mean ± SD (*n* = 5)) in methanolic extracts (methanol/water, 1:1, *v*/*v*), using the standard Folin–Ciocalteu method. Free radical scavenging capacity of the extracts was determined by ABTS^●+^ and the DPPH^●^ assays. The AA of wormwood EOs and extracts tested by the spectroscopic method is presented in Figure 3 and Table A1 (Appendix A).

The ABTS^●+^ scavenging assay of wormwood EO yielded negligible activity (close to 0), likely due to its limited solubility in the assay medium. Therefore, corresponding results were not included and not considered for further interpretation.

### 2.4. AA of A. absinthium Extracts Tested by Differential Pulse Voltammetry (DPV)

For all electrochemical experiments, aqueous extracts (infusions) were used to ensure compatibility with the electrochemical system, to reflect the conditions relevant to practical applications, and to avoid the potential impact of organic solvents.

Carbon paste electrodes were prepared by combining graphite powder with a water-immiscible, non-electrolytic organic binder. They provided reliable analytical performance over a broad potential range, characterized by low background currents, minimal ohmic resistance, and flexibility in adjusting pretreatment procedures and surface modifications. Additionally, these electrodes are environmentally benign and cost-effective. However, their main limitation is surface irregularities, which may affect the reproducibility of the obtained signals [64].

**Figure 4:**
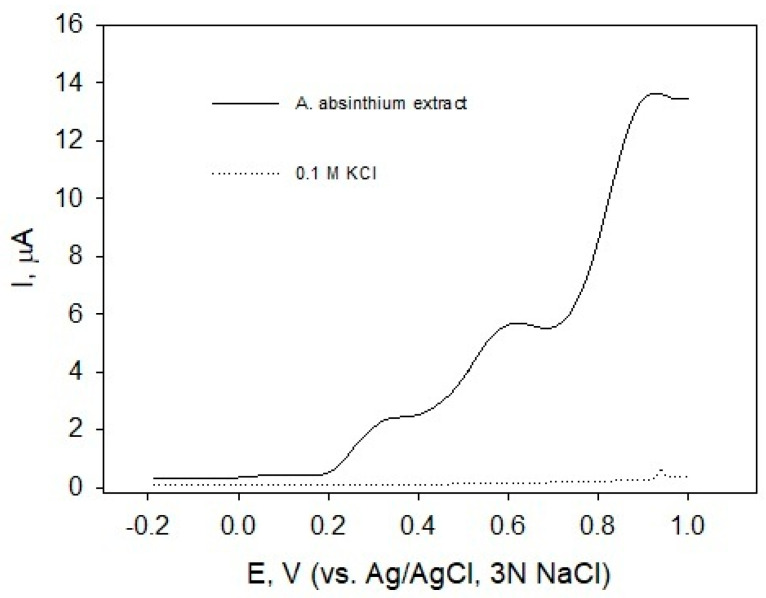
Differential pulse voltammograms of carbon paste electrode of *A. absinthium* water extracts containing 0.1 M KCl; potential step 4 mV, pulse width 50 ms, pulse period 200 ms, pulse amplitude 50 mV.

The carbon paste working electrode was immersed in the sample solution, and its potential was scanned in the positive direction. During this forward scan, the electrode potential progressively increased, enhancing its oxidizing power. Once the applied potential reached the oxidation potential of an electroactive species, oxidation occurred. Compounds that oxidize at lower potentials exhibited stronger reducing (antioxidant) properties. The anodic peak potential (E_pa_) is influenced by factors, such as the electroactive substance’s chemical structure, the electrode material, and the solution’s composition and pH. In contrast, the anodic peak current (I_pa_) at a given E_pa_ reflected the concentration of the electroactive species. Differential pulse voltammetry (Figure 4) revealed relatively easily oxidizable substances in the aqueous extract of *A. absinthium*, with E_pa_ values observed at approximately 0.38 and 0.61 V (solid line).

### 2.5. Hydrogen Peroxide (H_2_O_2_) Scavenging by A. absinthium Aqueous Solutions

**Figure 5:**
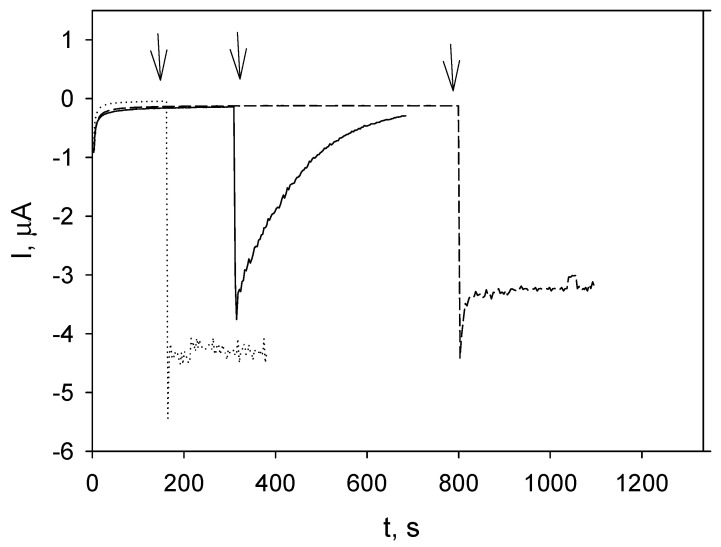
Responses of MC/PB in phosphate buffer pH = 6 (dotted line), *A. absinthium* aqueous extract (solid line) and *A. absinthium* infusion (dashed line) to the additions of H_2_O_2_, operating potential 0.0 V. Arrows indicate the moments of H_2_O_2_ addition.

Prussian blue (PB) (iron(III) hexacyanoferrate(II)) pigment has a specific electrocatalytic properties which allow it to reduce hydrogen peroxide at potentials around 0.0 V, excluding the influence of other electroactive species [65]. PB has the ability to form a coordination polymer; thus, the PB-modified electrode (MC/PB) was prepared. Initially, the electrode was kept at a constant potential of 0.0 V until the background current attained a stable state. Subsequently, a steady cathodic current associated with the H_2_O_2_ reduction at the MC/PB electrode was detected (Figure 5, dotted line) following the injection of hydrogen peroxide into the phosphate buffer.

After addition of hydrogen peroxide to the tested *A. absinthium* extracts, the hydrogen peroxide-induced reduction current decreased rapidly. Within approximately five to eight min, the signal returned to its initial steady-state level (Figure 5, solid line shown for *A. absinthium* extract). The decline and eventual disappearance of the reduction current indicated that there was no electroactive substance, i.e., hydrogen peroxide was scavenged by the extracts. As demonstrated in Figure 5 (dashed line shown for *A. absinthium* infusion), after hydrogen peroxide injection into the wormwood infusions, steady cathodic currents were recorded, thereby demonstrating that infusions were incapable of H_2_O_2_ scavenging.

### 2.6. Prooxidant Activity of A. absinthium Extracts

In order to evaluate the prooxidant activity of aqueous extracts and infusions of wormwood, the production of hydrogen peroxide (H_2_O_2_) was tested in acidic and slightly alkaline medium, at pH ≈ 5.0 and pH = 7.2, respectively. As demonstrated in Table 3, the average value and standard deviation of the quantity of hydrogen peroxide generated within a 30 min time period was calculated.

## 3. Discussion

*A. absinthium* EOs obtained from aerial parts of the plant have a dark green to orange or dark brown color (sensitive to ultraviolet/visible light) and a strong specific odor. Sabinene, myrcene, pinenes, camphor, borneol, thujones, *epoxy*-ocimenes, bornyl or chrysanthenyl acetate, chrysanthenol, linalool and its oxide, 1,8-cineole, caryophyllene, chamazulene, guaiol, oleic acid, etc., have been identified as the most characteristic components of wormwood EOs previously [2,3,4,5,6,7,8,9,11,12,20,21,22,23,24,25,26,27,28,29,32,35,36,39,40,42]. In the wormwood EOs under the study, *trans*-sabinyl acetate (59.6%) was found to be the predominant constituent, and the oil could be attributed to the sabinyl acetate chemotype. Compounds of sabinene carbon skeleton (*α*-thujene, sabinene, *cis*-sabinene hydrate, *trans*-thujone, *trans*-sabinol, *α*-thuj-3-en-10-al and *trans*-sabinyl acetate) comprising 68.5% of total quantity were found to be the major faction in the EO (Table 1).

It should be mentioned that this monoterpenoid, sabinyl acetate has previously been determined in appreciable amounts in wormwood EOs [4,20,21,24,25,29,39,40,55,66,67]. *cis*-Sabinyl acetate (32.8%) together with *trans*-thujone (33.1%) were determined as the main constituents in the wormwood oils from the USA ([20] and therein cited). Wormwood EOs of the plants grown in Western Canada were characterized by high quantities of *trans*-sabinyl acetate (26.4%), myrcene (10.8%) and *trans*-thujone (10.1%) [66]. Major constituents, *cis*-sabinyl acetate (38.5%) and *cis*-epoxy-ocimene (28.8%), and relatively small amounts of thujones (4.0%) were isolated from the plants collected in Croatia [67]. Appreciable amounts of sabinyl acetate were detected in Estonian and French wormwood EOs (70.5 and 84.5%, respectively), and insignificant contents of *β*-thujone were found (2.3 and 0.7%, respectively) in the oils by Orav et al. [29]. However, the wormwood oils of the plants grown in Germany and Moldova were characterized by low quantities of thujone isomers in the above study [29]. The oils of *A. absinthium* of Italian and French origin contained epoxy ocimenes (56.6 and ≤49.7%, respectively) and no thujone isomers [29,40]. *trans*-Sabinyl acetate (55.2%) and the absence of thujones were found in Lithuanian wormwood oils [21]. Another Lithuanian wormwood oil was characterized by appreciable amount of *trans*-sabinyl acetate (45.2%) and (*cis* + *trans*) thujones (12.3%) [25].

*Trans*-sabinyl acetate is a valuable secondary metabolite exhibiting various pharmacological and toxic activities [25,27,66,67,68,69,70]. There is rather limited literature data regarding the bioactivity of sabinyl acetate or EOs with high concentrations of this monoterpenoid. Strong toxicity was revealed against brine shrimp (*Artemia* sp.) nauplii for Lithuanian wormwood oils in the above study [25]. The Canadian wormwood oils rich in *trans*-sabinyl acetate (26.4%) and *trans*-thujone (10.1%) demonstrated inhibitory effects on the growth of bacteria (*E. coli*, *S. aureus*, and *S. epidermidis*), yeasts (*C. albicans*, *C. neoformans*), dermatophytes (*T. rubrum*, *M. canis*, and *M. gypseum*), *Fonsecaea pedrosoi* and *Aspergillus niger,* while a weak antioxidant (beta-carotene/linoleate model and DPPH radical scavenging) activity was determined for these oils [66]. The Croatian *A. absinthium* oils containing major constituents, *cis*-sabinyl acetate and *cis*-epoxy ocimene exhibited acetylcholinesterase and butyrylcholinesterase effects [67]. Acetylcholinesterase inhibitory and in vivo antinociceptive activity as well as an acute toxicity of *trans*-sabinyl acetate was evaluated by Radulović et al. [68]. However, the embryo–fetotoxic, abortifacient and hepatotoxic properties were revealed for various EOs containing appreciable amounts of *trans*-sabinyl acetate [70]. Strong biological effects make the compound of interest in pharmaceutical and agricultural applications. Moreover, sabinyl acetate play a number of specific biological roles, including those relating to plant-insect interactions.

Eighteen phenolic acids were identified in the wormwood methanolic extracts (Table 2). Most of the acids found in this study were also reported previously [5,6,7,8,10,13,14,16,23,24,33,37,51,67,68,69]. It should be mentioned that common flavonoids present in *A. absinthium* extracts such as quercetin, kaempferol, apigenin, naringenin or luteolin [9,10,14,23,24,33,37,51] have not been determined (or in quantities below detection limits) in wormwood extracts under this investigation (Table 2). Also, it could be explained by their neglectable concentrations, comparing to the other constituents present in the extracts.

Antioxidant activity (AA) tests results are presented in Figure 3. AA is one of the characteristics that allows evaluating the pharmacological properties of the plants. Most of the phenolic acids, sesquiterpenoids, catechins and other phenolic compounds (mainly flavonoids) determined in the extracts in this study can exhibit stronger or weaker an oxidant activity. Usually, AA is studied by spectrophotometric methods, such as assays measuring the reducing effect (i.e., TROLOX equivalent antioxidant capacity (TEAC) and ferric reducing antioxidant power (FRAP)), radicals scavenging ability (i.e., DPPH and ABTS), and metal-chelating activity (MCA). Despite the fact that the results of antioxidant assays in the literature are not always properly comparable due to the different forms in which these data are presented, numerous literature sources indicate that various extracts and EOs (of wide compositional variability) of *A. absinthium* [6,7,9,10,12,16,20,22,30,31,33,35,38,39,43,47,48,51,52,53] possess a significant AA. AA tests are usually complemented with TPC, and very often a positive correlation is found between these two variables. Consequently, if a substantial free radical scavenging capacity has been identified, it is generally presumed that the plant extracts are abundant in phenolic compounds. It is well documented that the content of polyphenols in plant extracts is dependent on a variety of factors, including the method of preparation, the temperature of the procedure, the solvent utilized, the conditions of the herbal material, etc. [71,72,73,74].

The employment of electrochemical techniques has proven to be a highly efficacious technique for the evaluation of antioxidating potential of beverages, plant extracts, or individual polyphenols [64,75,76,77,78,79]. Electrochemical approaches are predicated on the physical–chemical properties of the compounds and can therefore be considered as a direct test for antioxidant properties. It is evident, that the electrochemical method (DPV) applied in our research is not frequently used to investigate antioxidant/prooxidant properties of EOs and plants extracts, and even more, there is no corresponding data related to *A. absinthium*. Voltammetric profiles (Figure 4) revealed that the extracts contained a compound characterized by E_pa_ at 0.38 V, and voltammograms of *A. absinthium* (Figure 4, solid line) clearly showed the presence of another relatively easily oxidizable compound with E_pa_ at 0.61 V. Different I_pa_ values at these potentials indicate different concentrations of certain extracted polyphenols. Polyphenols with relatively low and pH-dependent oxidation potentials are probably compounds with a flavonoid structure containing catechol or galloyl moieties [80]. A direct comparison of the obtained E_pa_ values with those found in the existing literature is difficult by the fact that the experimental conditions (electrode material, ionic strength of the solution, the presence of organic solvent, and concentration of electroactive substance, etc.) can cause peak shifts.

Electrochemical test showed that wormwood extracts, prepared at ambient temperature possessed a capability to scavenge hydrogen peroxide (Figure 5, solid line shown for *A. absinthium* extract). The polyphenol concentration was not critical as well as it was formerly observed for various *Rubus idaeus* extracts [81]. In the case of infusions, the reduction currents remained stable (Figure 5, dashed line, shown for *A. absinthium* extract), indicating the presence of hydrogen peroxide in the solution; i.e., the infusions did not scavenge peroxide. Similarly, unfavorable effect of elevated temperature was observed for *R*. *idaeus* decoctions [81]. An investigation of the enzyme and polyphenol fractions of *R. idaeus* bark extract confirmed that protein components, likely of an enzymatic nature, contribute to peroxide scavenging potential. The loss or absence of this activity in wormwood infusions may be due to the inactivation of specific enzymes. That was to be expected, especially since the extracts from many other plant species have also demonstrated the ability to neutralize peroxides. As demonstrated in the preceding publication [63], the H_2_O_2_ scavenging potential, ascertained via the FOX method (oxidation of ferrous ions using xylenol orange), exhibited no evident correlation with the phenolic content found within the plant extracts. Furthermore, it was observed that the activity was notably higher in acidic medium compared to alkaline one. In the context of alkaline pH conditions, the plant extracts can generate a certain quantity of H_2_O_2_ which was found to be dependent upon their phenolic content [63]. Moreover, the results of both antioxidant and prooxidant activity tests conducted by FRAP technique, showed that compounds with a higher number of oxygen heteroatoms (e.g., containing keto groups), such as flavonoids demonstrated prooxidant effects, and in contrast, catechins and carotenoids exhibited dominant antioxidant activity over prooxidant effects. [82]. In another study [83], the ability to produce hydrogen peroxide of plant extracts and phenolic compounds (acids and flavonoids) was examined using the FOX assay, and it was revealed that some plants extracts possessing an excellent combination of antioxidant and prooxidant activities, can also exhibit cytotoxic effects on cancer cells. Additionally, it was discovered that only chlorogenic acid, catechin, and quercetin can function both as antioxidants and prooxidants in the above study [83]. There is a definitive lack of already published papers on the potential of *A. absinthium* plant extracts to scavenge hydrogen peroxide, and no available data by electrochemical methods.

The amount of formed hydrogen peroxide was similar in the appropriate extracts (ultrasound-assisted or infusions) depending mainly on pH values (Table 3). The highest content of peroxide (31.86 ± 0.1 μmol/L) was determined for investigated infusions at pH = 7.2. It is well known, that hydrogen peroxide has bactericidal and virucidal effects, and could be used to treat acne, common warts and to care wounds [84]. Moreover, H_2_O_2_ is a physiologically relevant compound; it is effective against pathogenic microorganisms (at concentrations from µM to mM) and participates in intra- and inter-cellular signaling at nanomolar up to low micromolar concentrations [85,86,87,88]. Human’s every day consumption of peroxide is caused by beverages, such as tea, coffee and wines, and vegetables [82,85]. The hydrogen peroxide concentration in freshly brewed coffee can vary from 20 to 80 µM; in green and black tea, it can exceed up to 700 µM [84,85]. The presence of polyphenolic acids, i.e., caffeic, chlorogenic and gallic acids, as well as rutin and (epi)catechins in the extracts (Table 1), has been identified as a potential factor in the formation of hydrogen peroxide. Obtained data are in agreement with results in the literature [82,83].

Interpretation of the data obtained from the extracts would suggest speculatively that there is a negative correlation between TPC (in 50% methanol) and quantity of hydrogen peroxide (in aqueous solutions). There is a lot of scientific evidence that the qualitative and quantitative extraction of biologically active compounds is strongly influenced by the nature of the solvents and extraction conditions.

## 4. Materials and Methods

### 4.1. Plant Material

The aerial parts (~20 cm, up to 1.5 kg) of *Artemisia absinthium* L. from wild populations in Lithuania (North-Eastern part, Rokiškis district, Aleknos village, around 56°06′23.7″ N 25°39′34.6) were collected at the full flowering stage in 2021–2023. The investigated wormwood natural population is indicated in Figure S1. The raw material (comprising primarily leaves and flowers, up to 20 cm in length) was expeditiously transferred to the laboratory, where it was subjected to drying at room temperature (23 ± 2 °C) in a shaded and well-ventilated environment for a period of two weeks. Humidity of herbal material was measured using a Traceable ^®^ Thermometer/Clock/Humidity Meter (8709 Pen type hydrometer, Fisher Scientific, Webster, TX, USA). The characteristic specimen of *A. absinthium* L., approved by Dr. M. Rasimavičius, has been placed in the Vilnius University Herbarium (Lithuania) under the code number P33734.

### 4.2. EO Isolation from A. absinthium L. Plants

In accordance with the European Pharmacopoeia [89], the essential oils from aerial parts (leaves and flowering tops) of air-dried plants (80–100 g) were extracted by hydro-distillation (2 h) procedure in a Clevenger-type apparatus. The volume ratio of plant material to water was 1:20. The obtained EOs were immediately subjected to desiccation over dried over anhydrous Na_2_SO_4_, then were transferred to hermetic dark vials and deposited in a refrigerator (set at −18 °C). Prior to analysis, the samples were dissolved in a mixture (1:1, Vol:Vol) of pentane and diethyl ether. Pentane and sodium sulphate (produced in India) were purchased from Sigma Aldrich Co. (St. Louis, MO, USA), diethyl ether from C. Roth GmbH + Co. (Karlsruhe, Germany).

### 4.3. Preparation of Various A. absinthium Extracts for Chemical Analysis

#### 4.3.1. Preparation of A. absinthium Extracts for HPLC/DAD/TOF Analysis

Samples of air-dried herbal material (wormwood inflorescences and leaves) were prepared according to the procedure described previously [90]. The obtained mixture was filtered through a filter paper (pore size 11 µm (Whatman, GmbH, Germany), and then by nylon syringe filters (0.22 mm) before analysis. Methanol was purchased from Honeywell (Seelze, Hanover, Germany).

#### 4.3.2. Preparation of A. absinthium Extracts for TPC and Free Radical Scavenging Capacity Measurements

In total, 2.5 g of dry crushed plant material (flowers and leaves) was poured with 25 mL of distilled water and extracted in an ultrasonic bath for 30 min. The mixture was filtered and then subjected to spectrophotometric analyses for TPC and free radical scavenging capacity determination.

#### 4.3.3. Extraction Procedure for AA Tests by Electrochemical Measurements

In total, 5 g of ground wormwood inflorescence and leaf powder was placed in 75 mL of 0.1 M KCl (for DPV) or pH 6.0 (for H_2_O_2_-scavenging test) consisting of 0.05 mM KH_2_PO_4_ and 0.1 M KCl (both from Fluka, Sigma Aldrich Chemie GMbH, Steinheim, Germany). The pH value was adjusted with KOH (Fluka). Extractions were performed with ultrasound for 30 min. The extracts were filtered through a filter paper. Infusions were prepared by pouring of 75 mL of boiling phosphate buffer onto 5 g of ground *A. absinthium* material and allowed to cool to room temperature (23 ± 2 °C).

### 4.4. GC (GC/FID and GC/MS) Analysis of A. absinthium EOs

Quantitative analyses of the wormwood EOs (at least 3 repetitions per analysis) were carried out on Perkin-Elmer Clarus 500 chromatograph equipped with a FID (Flame-Ionization Detector) (Hewlett Packard, Palo Alto, CA, USA), using DB-5 ((5%–phenyl)-methylpolysiloxane; 50 m × 0.32 mm i.d., film thickness 0.25 μm) and DB-Wax (30 m × 0.25 mm, film thickness 0.25 μm) capillary columns (Agilent, J&W Scientific, Santa Clara, CA, USA). The temperature conditions of GC oven, parameters of injector, detector and carrier gas were identical as in our previous research [90].

Qualitative analyses were performed on a chromatograph Shimadzu GC-2010 PLUS (Shimadzu, Kyoto, Japan) interfaced to a Shimadzu GC-MS-QP2010 ULTRA mass spectrometer (Shimadzu, Kyoto, Japan) and fitted with a capillary column Rxi-5MS ((5%–phenyl)-methyl polysiloxane, 33 m × 0.25 mm i.d., film thickness 0.25 µm, Restek, Bellefonte, PA, USA). The working parameters of mass spectrometer and ion source, and the conditions of chromatographic separations and the method of identification of individual components were the same as in our previous research [90]. Additionally, some standards (sabinene, *β*-myrcene, *α*-, *β*-pinene, *p*-cymene, linalool, *trans*-thujone, (2*E*,6*E*)-farnesol, *β*-caryophyllene and its oxide) purchased from Sigma Aldrich (Darmstadt, Germany) and Honeywell Fluka (Fisher Scientific, Loughborough, Leicestershire, UK) were applied for identification of individual constituents in the wormwood EO.

### 4.5. HPLC/DAD/MS (TOF) Analysis of A. absinthium Extracts

Extracts (prepared according to chapter 4.3.1) from inflorescences and leaves were analyzed by HPLC technique using a system HPLC/Diode Array Detector (DAD)/Time of Flight (TOF) (Agilent 1260 Infinity (Agilent Technologies, Waldbronn, Germany) and Agilent 6224 TOF (Agilent Technologies, Santa Clara, CA, USA) equipped with a reverse phase column ZORBAX Eclipse XDB (C18, 5 μm particle size, 150 × 4.6 mm, Agilent Technologies, Santa Clara, CA, USA). The column temperature, eluents composition, chromatographic separation, TOF acquisition and ionization parameters were the same as in paper [90]. An injected samples volume varied from 5 to 15 μL. Standards of main phenolic acids (fumaric, ascorbic, gallic, *trans*-ferulic, *p*-hydroxybenzoic, chlorogenic, etc.) with purity ≥ 95–99% were purchased from Merck and Sigma-Aldrich Solutions (Darmstadt, Germany). Standard of rutin (97+%) was from Acros Organics (Geel, Belgium) and of flavanols, (+)-catechin hydrate and (-)-epicatechin was received from Fisher Scientific (Loughborough, Leicestershire, UK). References of flavonoids, quercetin-3-*O*-glucoside and apigenin-7-glucoside were bought from Merck and Sigma-Aldrich Solutions (Darmstadt, Germany). Acetonitrile was purchased from Honeywell (Seelze, Hanover, Germany), and formic acid from Sigma Aldrich Co. (St. Louis, MO, USA).

### 4.6. Determination of TPC in A. absinthium Extracts

TPC was determined in wormwood extracts by the Folin–Ciocalteu assay [91], and the measurements were performed using the spectrophotometer (UV/Vis C-7200S, PEAK Instruments Inc., Houston, TX, USA) [90]. The calibration curve was drawn using different concentrations of gallic acid (Figure S2 in Supplementary Materials). All measurements were performed with five replicates.

### 4.7. Spectrophotometric ABTS^●+^ and DPPH^●^ Scavenging Assays

**Figure 6:**
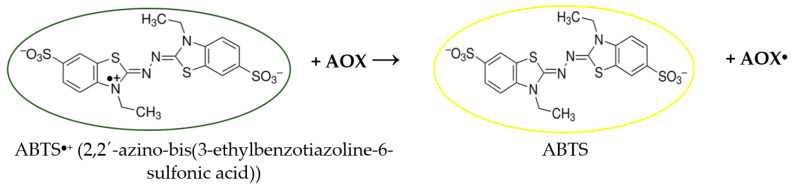
Schematic presentation of ABTS assay.

The AA of wormwood EOs and extracts was tested by the spectroscopic method described in the literature [81,90,92]. Scheme of ABTS radical scavenging test is presented in Figure 6.

**Figure 7:**
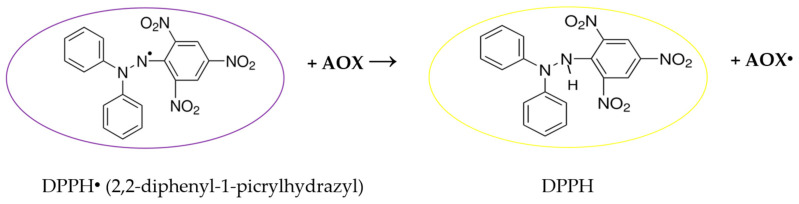
Schematic presentation of DPPH assay.

Wormwood EOs and extracts for analysis were diluted 1:50 with a mixture of methanol and water (80:20); 0.1 mL of prepared sample was allowed to react with 3.9 mL of working ABTS^●+^ solution for 15 min in the darkness. The results were expressed in mmol/L TROLOX equivalent. All measurements were performed with five replicates. Scheme of DPPH radical scavenging test is presented in Figure 7.

### 4.8. TROLOX Equivalent ABTS^●+^ and DPPH^●^ Assays

Five mg of TROLOX (±)-6-hydroxy-2,5,7,8-tetra-methylchromane-2-carboxylic acid) was dissolved in a methanol/water solution (70:30) and diluted to 100 mL. Five different concentrations (200, 100, 50, 25 and 12.5 mmol/L) from this solution were prepared. A total of 0.1 mL of each TROLOX solution was allowed to react with 3.9 mL of working solution of ABTS**^●^**^+^ or DPPH**^●^**. The absorbance was measured using the spectrophotometer (UV/Vis C-7200S, PEAK Instruments Inc., Houston, TX, USA) after 15 and 30 min at 734 and 515 nm, respectively. Obtained linear calibration curves and their parameters were used for further calculations of antioxidant capacity. All measurements were done in triplicate.

### 4.9. Electrochemical Tests

#### 4.9.1. Differential Pulse Voltammetry (DPV)

Details of amperometric measurements performed in our laboratory, using the BAS-Epsilon Bioanalytical system (West Lafayette, IN, USA), were presented in the literature [81]. A conventional three-electrode cell contained a carbon paste electrode as a working electrode, platinum as an auxiliary electrode, and Ag/AgCl, 3 N NaCl as a reference electrode. The carbon paste electrode was prepared by thoroughly mixing 200 mg of graphite powder with 100 µL of paraffin oil. The paste was packed into the cavity of a homemade electrode consisting of a plastic tube (2.9 mm) and a copper wire serving as an electrode contact. The surface of the electrode was thereafter smoothened on a white paper.

Conditions of DPV at carbon paste electrode, applied for *A. absinthium* leaf and flower extracts in aqueous 0.1 M KCl, were already described [90].

#### 4.9.2. Hydrogen Peroxide Scavenging Test

The tests, using the Prussian Blue-modified electrode (MC/PB) were performed according to the method documented in our previous research [81].

### 4.10. Prooxidant Activity Test

The tests of *A. absinthium* inflorescence and leave extracts and infusions prepared in 0.1 M KCl (pH ≈ 5.0) and phosphatic buffer (pH = 7.2) were done with FOX reagent (250 μM FeSO_4_, 25 mM H_2_SO_4_, 100 μM xylenol orange and 100 mM sorbitol) according to the method described in the paper [81]. The absorbance of the solution was measured at 580 nm wavelength and calibration curves (Figures S3 and S4 in Supplementary Material) were created using 1 to 100 μM hydrogen peroxide solutions.

## 5. Statistical Analysis

The obtained experimental data were statistically processed, using XLSTAT (trial version, Addinsoft 2014, Paris, France) and IBM SPSS Statistics software (Version 28.0.1.1(15), New York, NY, USA) [81].

## 6. Conclusions

The present research makes a contribution to the knowledge of the phytochemical profile and pharmacological properties (antioxidant/prooxidant) of *Artemisia absinthium* L. collected from spontaneous Lithuanian flora. On a basis of obtained data, further propagation and industrial cultivation of the plants could be possible. The presence of toxic and neurotoxic thujone is undesirable in many cases, because of that, searching for low or thujone-free plants from natural habitats and possessing pharmacological properties is very important. It should be mentioned that there is a lack of available data related to wormwood plants synthesizing EOs containing low amounts of thujones and high concentrations of *trans*-sabinyl acetate. The low content of toxic thujone isomers and the high quantity of *trans*-sabinyl acetate (59.6%) in wormwood EOs make them in demand for various needs and applications. Our research has filled a gap in the literature regarding biological studies of EOs of *trans*-sabinyl acetate chemotype. A list of various phenolic compounds, such as phenolic acids and flavonoids present in *A. absinthium* extracts was documented. Total phenolic content was found 412.82 ± 11.10 mg/L (of gallic acid equivalent) in *A. absinthium* methanolic extracts. A positive correlation was observed between TPC (mainly due to the polyphenolic compounds present in the *A. absinthium* extracts) and strong ability of the extracts to scavenge free radicals. Values of ABTS^●+^ and DPPH^●^ scavenging capacity were determined to be 3.49 ± 0.07 and 6.48 ± 0.25, (mmol/L TROLOX equivalent), respectively. On the contrary, AA of wormwood EO was weaker (0.83 ± 0.06 mmol/L, TROLOX). DPV revealed the presence of electrochemically oxidizable compound(s) with characteristic E_pa_ values of 0.38 V and 0.61 V. The lower the E_pa_ value, the higher antioxidant activity of the compound(s). Electrochemical hydrogen peroxide scavenging test showed that the activity did not depend on TPC. Hydrogen peroxide formation was observed in the aqueous extracts, and H_2_O_2_ quantity was mainly pH-dependent with a weak effect of temperature. The largest quantity of peroxide (31.86 ± 0.1 μmol/L) was formed in the wormwood boiling infusions (at pH = 7.2).

Further investigations into wild-growing medicinal plants that produce desirable secondary metabolites for propagation purposes are promising.

## Figures and Tables

**Table 1:** Chemical composition (≥0.05%, values were expressed as means ± SD (*n* = 3)) of *A. absinthium* L. (plants collected in Rokiškis district, Lithuania (2021–2023)) EOs of inflorescences and leaves.

Compound ^a^	^b^ RI_Exp_ Rxi-5MS	^c^ RI_Exp_ DB-Wax	Percentage
^MH^*α*-Thujene	933	1020	0.1 ± 0.0
^MH^*α*-Pinene *	938	1022	0.4 ± 0.3
^MH^ Camphene	955	1059	0.2 ± 0.1
^MH^ Sabinene *	978	1116	1.2 ± 0.6
^MH^*β*-Pinene *	989	1098	0.2 ± 0.1
^MH^*β*-Myrcene *	991	1166	0.5 ± 0.2
^MH^*δ*-2-Carene	1004	1144	0.2 ± 0.1
^MH^*p*-Cymene *	1024	1269	0.5 ± 0.3
^OxM^ 1,8-Cineole	1033	1213	0.7 ± 0.3
^MH^*γ*-Terpinene	1064	1240	0.5 ± 0.1
^OxM^*cis*-Sabinene hydrate	1068	1481	0.3 ± 0.1
^MH^*α*-Terpinolene	1088	1274	0.7 ± 0.3
^OxM^ Linalool *	1096	1555	1.2 ± 1.0
^OxM^*trans*-Thujone *	1114	1448	1.2 ± 0.5
^OxM^*trans*-Sabinol	1142	1711	5.9 ± 2.1
^OxM^*trans*-Verbenol	1146	1674	0.2 ± 0.2
^OxM^ Isoborneol	1158	1671	0.3 ± 0.2
^OxM^ Terpinen-4-ol	1179	1602	1.1 ± 0.7
^OxM^*α*-Thuj-3-en-10-al	1184	1640	0.2 ± 0.1
^OxM^*α*-Terpineol	1192	1688	0.5 ± 0.2
^OxM^ Geraniol	1255	1850	0.6 ± 0.4
^OxM^ Bornyl acetate	1285	1570	0.1 ± 0.1
^OxM^*trans*-Sabinyl acetate	1290	1625	59.6 ± 10.1
^SH^*β*-Bourbonene	1384	1528	0.6 ± 0.4
^SH^*trans*-*β*-Caryophyllene *	1419	1608	0.5 ± 0.1
^SH^*α*-Humulene	1455	1645	0.7 ± 0.3
^SH^*γ*-Curcumene	1485	1688	1.5 ± 0.6
^SH^ Viridiflorene	1496	1698	0.3 ± 0.1
^OxS^*trans*-Nerolidol	1563	2010	0.4 ± 0.1
^OxS^ Caryophyllene oxide *	1586	2008	0.9 ± 0.5
^OxS^ Humulene epoxide II	1608	2020	0.3 ± 0.1
^OxS^ Selin-11-en-4-ol	1655	2248	2.9 ± 0.9
^OxS^*epi*-*α*-Bisabolol	1684	2214	4.1 ± 1.1
^SH^ Chamazulene	1728	2370	0.7 ± 0.3
^OxS^ (2*E*,6*E*)-Farnesol *	1722	2365	0.3 ± 0.1
^OxS^*cis*-Nuciferol	1763		1.3 ± 0.7
^OxD^ Phytone	1855	2110	0.3 ± 0.1
^AH^*n*-Nonadecane *	1900		0.5 ± 0.3
^DH^ Geranyl-*α*-terpinene	1910	2145	3.5 ± 1.6
^AH^*n*-Eicosane *	2000		0.3 ± 0.1

^a^ Constituents are listed in order of their elution from a nonpolar Rxi-5MS column. Compounds are identified by their mass spectra and retention indices on both (polar DB-Wax and nonpolar Rxi-5MS) columns. ^b^ RI_Exp_: retention indices determined experimentally on the nonpolar column Rxi-5MS. ^c^ RI_Exp_: retention indices determined experimentally on the polar column DB-Wax. * Additional identification with reference compounds. Abbreviation of compound classes: ^MH^ Monoterpene hydrocarbon; ^OxM^ Oxygenated monoterpene; ^SH^ Sesquiterpene hydrocarbon; ^OxS^ Oxygenated sesquiterpene; ^DH^ Diterpene hydrocarbon; ^OxD^ Oxygenated diterpene and ^AH^ Aliphatic hydrocarbon.

**Table 2:** Tentative identification of compounds in methanolic *A. absinthium* inflorescence and leaf extracts analyzed by HPLC-DAD-TOF.

Compound	Formula	Molar weight	*m/z* ESI^+^ (Da)	*m/z* ESI^−^ (Da)
Acids:				
Fumaric *	C_4_H_4_O_4_	116.07	116.070	
4-Hydroxyphenylacetic	C_8_H_8_O_3_	152.15	153.023	
Ascorbic *	C_6_H_8_O_6_	176.13		175.118
Succinic *	C_4_H_6_O_4_	118.09	118.091	
Citric *	C_6_H_8_O_7_	192.12		191.011
Quinic	C_7_H_12_O_6_	192.17		191.017
Malic	C_4_H_6_O_5_	134.09		133.012
Gallic *	C_7_H_6_O_5_	170.12	171.134	
Benzoic *	C_7_H_6_O_2_	122.12	123.018	
Glutaric	C_5_H_8_O_4_	132.11	132.102	
Chlorogenic (3-*O*-caffeoylquinic acid) *	C_16_H_18_O_9_	354.31	355.101	353.084
Neochlorogenic (5-*O*-caffeoylquinic)	C_16_H_18_O_9_	354.31	355.101	353.084
Diferulic	C_20_H_18_O_8_	386.40	387.111	
*p*-Hydroxybenzoic (*p*-salicylic) *	C_7_H_6_O_3_	138.12	139.041	
Ferulic (hydroxycinnamic) *	C_10_H_10_O_4_	194.18	195.123	
Caffeic *	C_9_H_8_O_4_	180.16	181.082	
Isochlorogenic (3,5-dicaffeoylquinic) acid A	C_25_H_24_O_12_	516.45	517.134	515.118
Isochlorogenic (3,4-dicaffeoylquinic) acid B	C_25_H_24_O_12_	516.45	517.134	515.118
Other compounds:				
Epicatechin *	C_15_H_14_O_6_	290.27	291.087	
Catechin *	C_15_H_14_O_6_	290.26	291.094	
Quzhaqigan (piceatannol-3′-*O*-glucoside)	C_20_H_22_O_9_	406.4	407.242	
Diosmetin (5,7,3′-trihydroxy-4′-methoxyflavone)	C_16_H_12_O_6_	300.26	301.121	
Rutin *	C_27_H30O_16_	610.52	611.163	
Quercetin-3-*O*-glucoside *	C_21_H_19_O_12_	463.40	464.227	
Astragalin (kaempferol 3-*O*-glucoside)	C_21_H_20_O_11_	448.38		447.199
Apigenin-7-*O*-glucoside *	C_21_H_20_O_10_	432.38	433.279	
Hesperidin	C_28_H_34_O_15_	610.60	612.265	
Baicalin (7-D-glucuronic acid-5,6-dihydroxyflavone)	C_21_H_18_O_11_	446.40	447.201	
Quercetin 3-*O*-rhamnoside-7-*O*-glucoside	C_27_H_30_O_16_	610.50	612.267	
5,7,3′-Trihydroxy-3,6,4′,5′-tetramethoxyflavone	C_19_H_18_O_9_	390.30	391.283	

* Additional identification with reference compounds.

**Table 3:** The amount of hydrogen peroxide (μmol/L, mean ± SD, *n* = 5) in variuos *A. absinthium* water extracts and infusions produced in 30 min.

Wormwood Extracts	H_2_O_2_, μmol/L *
phosph. buffer, pH = 7.2, 30–35 °C, 30 min. ultrasound	26.89 ± 0.1 ^a, b, c^
infusion, boiling phosph. buffer pH = 7.2, allowed to cool to room temp. (23 ± 2 °C)	31.86 ± 0.1 ^a, b, c^
0.1 M KCl, pH ≈ 5.0, 30–35 °C, 30 min. ultrasound	2.68 ± 0.15 ^a, b, c^

* Means with significant difference are marked by letters (^a^, ^b^ and ^c^) (*p* < 0.05).

## Data Availability

The original contributions presented in this study are included in the Article/Supplementary Materials. Further inquiries can be directed to the corresponding author.

## Supplementary Materials

The following supporting information can be downloaded at: https://www.mdpi.com/article/10.3390/molecules31101551/s1, Figure S1: Geographical indication of sampling site of *Artemisia absinthium* L. in Lithuania (North-Eastern part, Panevėžys County, Rokiškis district, Aleknos village); Figure S2: Gallic acid standard calibration curve, using Folin–Ciocalteu method; Figure S3: Hydrogen peroxide standard calibration curve (range from 0 to 1000 µmol/L) and Figure S4: Hydrogen peroxide standard calibration curve (range from 0 to 120 µmol/L).

## Abbreviations

The following abbreviations are used in this manuscript:

HPLC/DAD/TOF	High-performance Liquid Chromatography/Diode Array Detector/Time of Flight Mass spectrometry
GC/MS	Gas chromatography/Mass spectrometry
DPV	Differential pulse voltammetry
PB	Prussian Blue
MC/PB	Prussian Blue-modified electrode
EO	Essential oil
TROLOX	6-Hydroxy-2,5,7,8-tetramethylchroman-2-carboxylic acid (C_14_H_18_O_4_)
ABTS	2,2’-Azino-bis(3-ethylbenzotiazoline-6-sulfonic acid) diammonium salt
DPPH	2,2-Diphenyl-1-picrylhydrazyl
GAE	Gallic acid equivalent
TPC	Total phenolic content
AA	Antioxidant activity

## Appendix A

**Table A1:** Antioxidant activity (AA) of *A. absinthium* EOs and extracts (obtained from flowering tops and leaves). Values were expressed as means ± SD (n = 3).

Scavenging Activity, TROLOX (mmol/L)	DPPH^●^	ABTS^●+^
Essential oil	0.83 ± 0.06	
Extract (MeOH/H_2_O, 1:1)	6.48 ± 0.25	3.49 ± 0.07
