# Supplementary material for: Low-Thujone A. absinthium L. (Wormwood) Essential Oils and Extracts with Potential Antioxidative/Prooxidant Activity
Source: Molecules. 2026 May 7;31(10):1551. doi: 10.3390/molecules31101551 (PMC13209449; doi:10.3390/molecules31101551)
Supplement: Supplementary file 1 [file molecules-31-01551-s001.zip › molecules-4232092-supplementary.pdf]

Low-Thujone *A. absinthium* L. (Wormwood) Essential Oils and Extracts with Potential Antioxidative/Prooxidant Activity

Asta Judžentienė and Jurga Būdienė

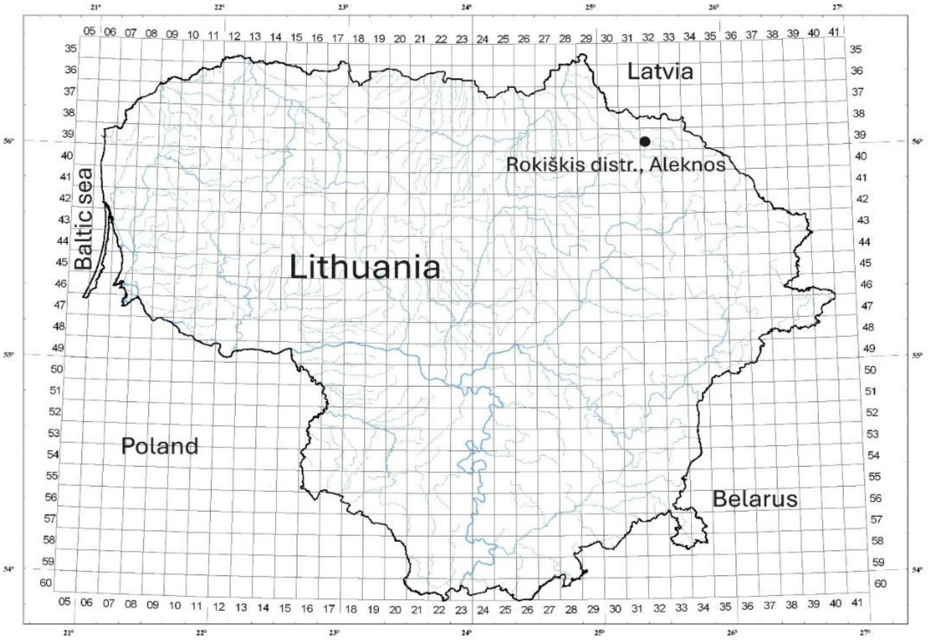

Figure S1. Geographical indication of sampling site of *Artemisia absinthium* L. in Lithuania (North-Eastern part, Panevėžys County, Rokiškis district, Aleknos village).

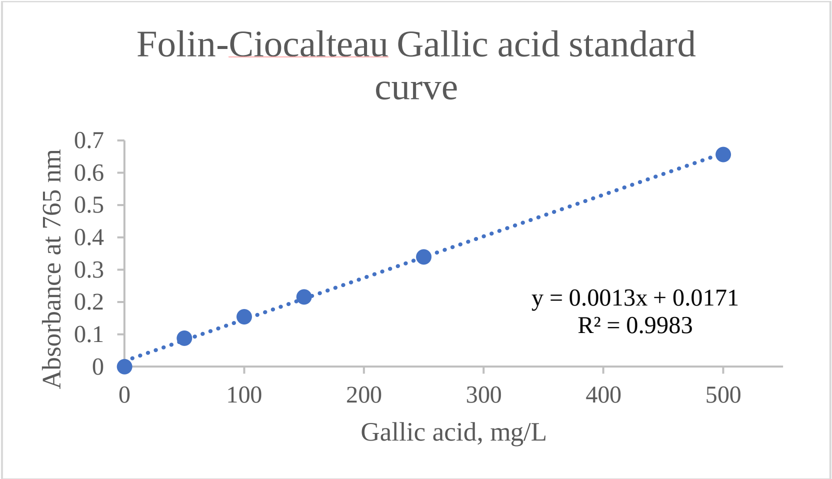

Figure S2. Gallic acid standard calibration curve, using Folin-Ciocalteu method.

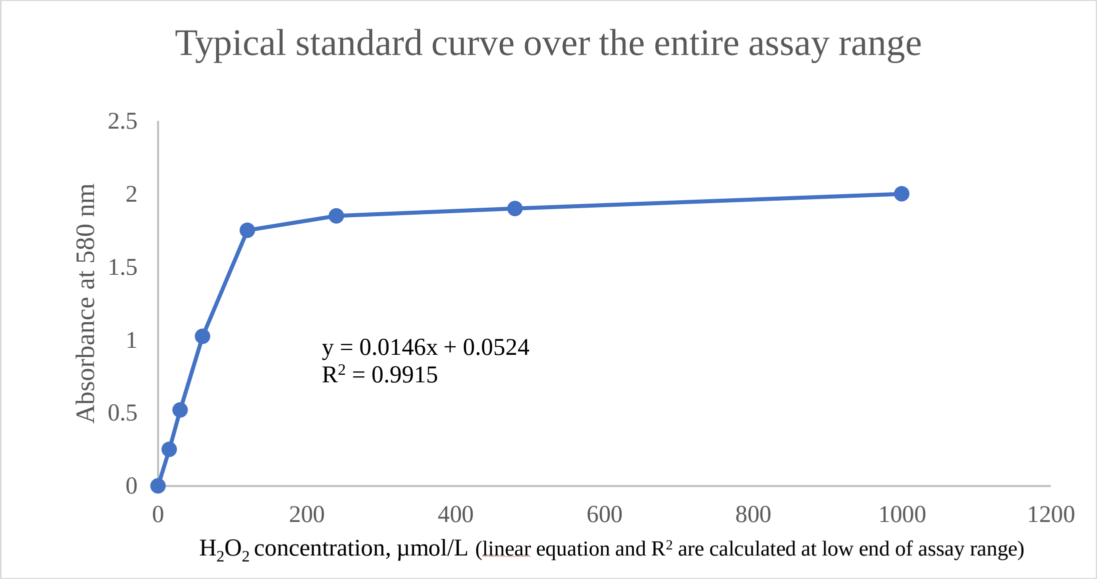

Figure S3. Hydrogen peroxide standard calibration curve (range from 0 to 1000 μmol/L).

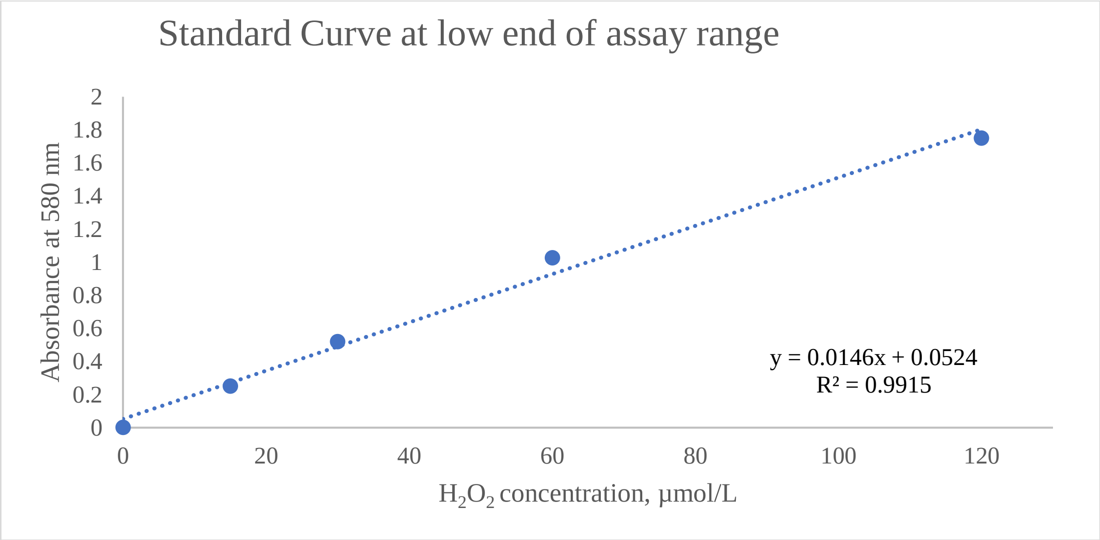

Figure S4. Hydrogen peroxide standard calibration curve (range from 0 to 120 μmol/L).
